# Multimodal Evoked Potentials as Potential Biomarkers of Disease Activity in Patients With Clinically Isolated Syndrome

**DOI:** 10.3389/fneur.2021.678035

**Published:** 2022-02-08

**Authors:** Edyta Dziadkowiak, Małgorzata Wieczorek, Mieszko Zagrajek, Justyna Chojdak-Łukasiewicz, Ewa Gruszka, Sławomir Budrewicz, Anna Pokryszko-Dragan

**Affiliations:** ^1^Department of Neurology, Wrocław Medical University, Wrocław, Poland; ^2^Faculty of Earth Sciences and Environmental Management, University of Wrocław, Wrocław, Poland

**Keywords:** multiple sclerosis, clinically isolated syndrome, multimodal evoked potentials, event-related potentials, cognitive performance

## Abstract

**Objective:**

There is an ongoing search for markers useful in monitoring and predicting disease activity at the early stage of multiple sclerosis (MS). The goals of this study were to prospectively evaluate the changes in parameters of multimodal evoked potentials (EP) and cognition within a 3-year follow-up period in patients with clinically isolated syndrome (CIS), and to assess the prognostic value of baseline findings with regard to the disease outcomes.

**Methods:**

In 29 patients (20 women, nine men, mean age 31.1) multimodal (visual, brainstem auditory, somatosensory, event-related) EP and neuropsychological tests (NT) were performed at baseline (T0) and after 1 (T1) and 3 (T3) years. Their results were compared longitudinally between baseline, T1, and T3. Baseline results confirmed conversion of CIS into multiple sclerosis (MS) and disability level at T1 and T3 using multiple comparisons and a logistic regression model.

**Results:**

Apart from mean N13/P16 SEP (somatosensory evoked potentials) amplitude (lower at T1 and T3 than at baseline (T0 1.02 ± 0.37 μV, T1 0.90 ± 0.26 μV, T3 0.74 ± 0.32 μV, *p* < 0.05 for both comparisons), no significant changes of EP or NT parameters were found in longitudinal assessment. Baseline P300 Pz latency was longer for the patients with MS than for those with CIS at T1 (352.69 vs. 325.56 ms). No predictive value was shown for any of the analyzed baseline variables with regard to conversion from CIS into MS.

**Significance:**

Baseline ERP abnormalities were associated with their short-term conversion into MS. ERP are worth considering in multimodal EP evaluation at the early stage of MS.

## Introduction

Clinically isolated syndrome (CIS) is defined as the first clinical episode, suggestive of multiple sclerosis (MS) (1). According to the current version of McDonald's criteria (1), some patients with CIS can be already diagnosed with MS, which allows clinicians to initiate early disease-modifying treatment (DMT). In those who do not fulfill the criteria for dissemination in time, a cautious follow-up is recommended, in order to recognize the development of active relapsing-remitting MS in a timely manner. On the other hand, in a multi-center European study (2), as much as 27% of patients remained with CIS without satisfying McDonald's criteria after 15 years of follow-up. Due to such a high variability of a further disease course, there is an ongoing search for predictive biomarkers, already applicable at this early stage, which would allow clinicians to stratify the risk of highly active MS and individualize therapeutic approaches (3). There is relevant evidence for the predictive value of magnetic resonance imaging (MRI) measures and presence of oligoclonal bands of Ig in cerebrospinal fluid (CSF), while the role of clinical issues, environmental factors (vitamin D deficiency, Epstein-Barr virus infection, smoking), and biochemical/immunological markers remains disputable (2–4).

Evoked potentials (EP), although recently degraded from McDonald's diagnostic criteria, provide a relevant measure of functional central nervous system (CNS) impairment and allow detection of subclinical neurological deficit in the course of MS (5). EP parameters were postulated to have some predictive value with regard to future disability (6–9) and conversion from CIS into clinically definite MS (10, 11). The application of multimodal EP corresponds with heterogeneity and dissemination in the space of MS-related CNS damage (12). Event-related potentials (ERP) are electrophysiological markers of cognitive performance, which is often present but underestimated at the earliest stage of MS. In our previous study (13), we found impaired memory and attention, as well as abnormal parameters of the P300 component of ERP in patients with CIS. These findings encouraged our further investigation and prospective observation of a study group, with the use of extended electrophysiological protocol.

The first goal of the present study was to prospectively evaluate the changes in parameters of multimodal EP and cognitive performance within a 3-year follow-up period of patients initially diagnosed with CIS. Our second goal was to attempt to assess the prognostic value of the baseline electrophysiological and neuropsychological findings, with regard to conversion from CIS to MS.

## Materials

Our previous study (13), focused on cognitive performance and ERP, comprised 44 patients diagnosed with CIS according to the 2010 version of McDonald's criteria (14) during their hospitalization in the Department of Neurology between June 2012 and May 2014. All these patients were invited to take part in the follow-up visits at 12 and 36 months after their baseline evaluation. A total of 34 subjects attended the visit after 1 year and 29 after 3 years (altogether 15 were lost to follow-up), and those 29 were finally included in this study. This group comprised 20 women and 9 men, aged 21–48 years (mean 31.01, SD 6.37). Table 1 presents clinical characteristics of the study group. Expanded Disability Status Scale (EDSS) (15) score ranged from 1.0 to 2.0 (median 1.5). The international consensus criteria for MS that incorporated MR criteria have reflected the increased role of MRI in the diagnostic process. In addition to periventricular lesions, juxtacortical, infratentorial, and spinal cord lesions are specifically included in the 2010 dissemination in space criteria. With these current criteria, the presence of lesions in any of two of these four locations meets the ‘dissemination in space' criteria (16). All the patients in the study group fulfill MR criteria of dissemination in space, and eight patients presented with at least one gadolinium-enhanced active lesion. However, lesions within the optic nerve are not considered relevant according to these criteria. Because of the relatively small size of the study group, we did not divide it into subgroups due to the listed localization of MR lesions for further analysis. We have only included a distinction between the presence of only supra-tentorial lesions and both supra- and infra-tentorial lesions. In 18 out of 29 subjects CSF was tested at baseline for the presence of oligoclonal bands of immunoglobulins and the result was positive in seven cases.

**Table 1:** The demographic and clinical characteristics of the study group.

	**Clinically isolated syndrome (CIS)** ***n* = 19**	**Multiple sclerosis (MS)** ***n* = 10**	**All patients** ***n* = 29**
Female/male (F/M)	12/7	8/2	20/9
Age (mean)	30.2 ± 5.2	32.8 ± 8.2	31.1 ± 6.4
	F: 30.8 ± 5.0	F: 33.5 ± 8.9	F: 31.2 ± 6.8
	M: 29.3 ± 5.8	M: 30 ± 4.3	M: 29.4 ± 5.3
Clinical presentation: Optic neuritis	8	2	10
Brainstem symptoms	2	4	6
Sensory disturbances	7	5	12
Pyramidal symptoms	2	2	4
Multi-system involvement	0	3	3
MR dissemination in time	0	8	8
Oligoclonal bands in CSF	0	7	7 (*n* = 18)
EDSS score 1.0	19	7	26
score 1.5	0	3	3

*F, female; M, male; MR, magnetic resonance; CSF, cerebro-spinal fluid; EDSS, Expanded Disability Status Scale*.

After retrospective analysis, based on the 2017 version of McDonald's criteria (1), 10 patients initially diagnosed with CIS were reclassified as having MS at baseline.

Concomitant diseases (including psychiatric disorders or history of substance abuse) were excluded in all the patients on the basis of their medical records.

The control group consisted of 50 healthy volunteers (without any past or current relevant medical history, without symptoms and signs of neurological deficit in basic screening assessment, and with no subjective cognitive complaints), matched to the studied group for age (mean 46.24 SD 15.89), sex (5 M, 45 F), and educational level.

The study protocol was approved by the Ethics Committee of Wroclaw Medical University. All the subjects provided written informed consent before they were included in the study.

## Methods

### Design of the Study

At baseline, all the patients with CIS and healthy controls had multimodal EP performed: visual (VEP), brainstem auditory (BAEP), somatosensory (SEP), and auditory event-related (ERP). In the group of patients, neurological examination and neuropsychological testing were also performed. The data on MRI and CSF findings were obtained from the medical records.

During the follow-up visits (after 1 year and 3 years) multimodal EP, neuropsychological testing, and clinical evaluation [with assessment of disability in EDSS (15)] were repeated in the eligible patients. Worsening of disability level was defined as an increase in EDSS score ≥ 0.5 points in comparison with the baseline assessment. The data were also collected on occurrence and severity of relapses, MRI findings, and therapies applied within the analyzed period of time. At each stage of follow-up, the diagnosis of CIS /MS was re-evaluated according to the 2017 version of McDonald's criteria (1).

### Multimodal EP

Multimodal EP were conducted using Viking Quest equipment (Viasys Healthcare Inc., Conshohocken, Pennsylvania, USA), following the International Federation of Clinical Neurophysiology (IFCN) guidelines (10–12). The session took place in a quiet and dimmed room at 22–24°C. Superficial Ag/AgCl electrodes were placed on the scalp, according to the international 10–20 scheme and fixed using adhesive-conductive paste. At least two runs of the stimulation were performed to obtain the repetitive averaged response for each modality. Latencies and amplitudes (“peak to peak”) were determined for relevant components.

VEP were induced by a structural checkerboard pattern emitted on the screen at a distance of 1 m. Each eye was stimulated successively at a frequency of 1.88 Hz. The recording electrode was placed in Oz, the reference one in Fz, and the ground electrode on the forearm. Overall, 75 responses were averaged within the frequency band 1–30 Hz, at the analysis time of 500 ms. The latencies of N75, P100, and N145 components, the relative P100 latency, and P100-N145 amplitude were assessed.

BAEP were obtained by application (via headphones) of “clicks” to the stimulated ear (duration of 0.1 ms, frequency of 20.3 Hz, and intensity of 65 dB above the hearing threshold), while background noise (35 dB above the hearing threshold) was applied to the other ear. Responses were recorded from A1/A2 referenced to Cz, with the ground electrode on the forearm. In total, 2.000 responses were averaged in the frequency band 150–3,000 Hz with an analysis time of 10 ms. Latencies of the I, III, and V components, I-III, III-V, and I-V interlatencies, and amplitudes of I and V were evaluated.

SEP from upper limbs were achieved by stimulation of the median nerve at the wrist level with electric impulses (duration 100 mcs, frequency 4.7 Hz). The recording electrodes were placed at Erb's point (referenced to the contralateral point), at the level of the C7 segment of the spinal cord (referenced to Fz), and over parietal areas (C3/P3 and C4/P4, referenced to Fz). The ground electrode was placed on the forearm. A total of 300 responses were averaged within the analysis time of 100 ms. Latencies for the following components were assessed: N9, N10, N13, P16, N20, and P22, with interpeak latencies N20-N13 (central conduction time), as well as amplitudes of N9/P10, N13/P16, and N20/P22.

For individual subjects, parameters of the studied EP components were initially determined for each stimulated side separately and then one mean value of latency and amplitude was calculated for further analysis.

ERP were obtained with auditory stimuli (intensity 70 dB, duration 200 ms), applied binaurally via headphones. The “oddball paradigm” was used, with target tones (2 kHz, 20% of each series) randomly scattered among the non-target ones (1 kHz, 80% of each series). The subjects were asked to silently count the target tones. The recording electrodes were placed in Fz, Cz, and Pz, with linked earlobes (A1/A2) as the reference electrode and a ground electrode on the forearm. At least 30 target trials were averaged in each run, with a frequency band of 0.30–70 Hz and analysis time of 1,000 ms. Latencies and amplitudes were measured for the P300 component.

### Neuropsychological Testing

The Brief Repeatable Battery of Neuropsychological Tests (BRBNT) (17) was used, which includes: the Selective Verbal Reminding Test (SVRT), Spatial Recall Test (SpaRT), Symbol Digit Modalities Test (SDMT), Paced Auditory Serial Additive Test (PASAT), and Word List Generation (WLG). The following domains are covered by BRBNT: verbal and visuospatial learning/memory, sustained and divided attention, speed of auditory information processing, verbal fluency and semantic executive functions.

The SVRT is based on a multiple-trial learning paradigm. A list of 12 words is read by the examiner and the subject is instructed to ultimately recall all the words in six consecutive trials. After each trial the examiner completes the missing words. After 15 min, the subject is asked to recall the list of words again. The result includes a total count of remembered words in the main and delayed part of the test.

During the SpaRT, the participant is shown a pattern of 10 checkers on the board for 10 s and then is asked to reproduce it. The procedure is repeated three times. After 15 min, the subject is asked to reproduce the pattern without seeing it. The results include the number of correctly placed checkers in the main and delayed part of the test.

For the SDMT, the participant substitutes numbers 1–9 for geometrical symbols, according to the provided key, within 90 s. The result is the number of correct matches.

During the PASAT, the subject listens to a series of digits read in 3 s intervals and is asked to add 60 consecutive pairs of them (each digit is added to the preceding one). The result is the number of correct responses.

For the WLG, the participant is asked to list as many nouns as possible belonging to a given category (“fruits and vegetables”) within 90 s. The result is the number of appropriate responses.

The EP session, BRBNT, and clinical evaluation took place on the same day, in the morning hours, in the same air-conditioned rooms for all the subjects. The baseline assessment was arranged within the 2 months following the diagnosis of CIS, and at least 4 weeks after corticosteroids were tapered. The follow-up visits after 1 year and 3 years were scheduled at least 4 weeks after the most recent relapse or after initiating or switching DMT, to reduce the impact of these events upon the findings.

EP results at baseline were compared between the patients and controls. The results of BRBNT referred to the normative values, as published by Boringa et al. (18), and classified as abnormal if the score was 1.5 SD below the age-adjusted norm. In the group of patients, results of EP and BRBNT, obtained at baseline (T0) and the follow-up visits (T1—after 1 year, T3—after 3 years) were analyzed in regard to disease activity, to answer the following questions:

- Were there significant changes in the studied parameters between the evaluated time points?
- Did the patients with CIS and those already fulfilling the criteria for MS after 1 year or 3 years of follow-up differ in baseline values of studied parameters?
- Were the baseline values of studied parameters predictive for the patients' progression in EDSS during the 3 years?
- Were baseline values of studied parameters predictive for disease activity during the 3 years of follow-up? Evidence of disease activity was determined using NEDA-3 status (no clinical relapses, no worsening of disability, and no MRI evidence of new/active lesions) (19).

### Statistical Analysis

For comparisons of variables between the studied groups (patients vs. controls, patients with CIS vs. MS), the Student's *t*-test (for variables with normal distribution and with the preserved homogeneity of variance) or the non-parametric Mann-Whitney *U* test (for variables without normal distribution) were used. Normality was checked with the Shapiro Wilk test. Because of the measuring schedule, the variables were dependent on time. Hence the ANOVA Friedman test was performed. It allows us to check the significance of the dependence of the variables on time. To assess the role of baseline neurophysiological and neuropsychological assessment in predicting conversion to MS and NEDA-3 status, logistic regression was used. The Spearman's rank correlation analysis was used to determine the dependence of variables on age.

Alpha = 0.05 was assumed as significant for all the tests. Where it was necessary, Bonferroni (type adjustment) correction was provided. It was required in order to compare the interacting variables. Analysis was performed using Statistica 12.0 software.

## Results

### Clinical Evaluation

During the first year, relapse occurred in 5 patients and new/active lesions in MRI were found in 10. Within the subsequent 2 years, relapses occurred in two subjects and progression in MRI lesions occurred in eight. According to McDonald's criteria (1), 13 patients fulfilled the criteria for MS and 16 remained in the CIS category at T1; at T3 these proportions were 18 and 11, respectively (Table 2).

**Table 2:** The clinical characteristics of the longitudinal assessment study group.

	**T1**	**T3**
Relapse	5 (17%)	2 (7%)
New lesion in MR	10 (34%)	8 (27%)
Both (relapse + new lesion in MR)	3 (10%)	2 (7%)
DMT	25 (86%)	21 (72%)

*MR, magnetic resonance; DMT, disease-modifying therapy (for multiple sclerosis)*.

The median EDSS score in the study group at T1 was 1.0 (range 1.0–2.0) and at T3–1.5 (range 1.0–3.0). At T3 compared to baseline, the EDSS score increased in eight patients and remained stable in 21 (*p* = 0.123).

Within the first year of the follow-up, DMT was instituted in 25 patients: interferon beta (IFN β) in 23 cases and glatiramer acetate in 2 cases Table 2). Within the subsequent 2 years, three patients resigned from DMT, one patient interrupted treatment because of pregnancy, in one subject the treatment was switched from IFN β to dimethylfumarate and in one, it was switched from IFN β to glatiramer acetate and then to fingolimod.

### Multimodal Evoked Potentials

#### Baseline Assessment

The P100 latency of VEP was significantly longer in patients than in controls (115.67 ± 11.62 vs. 100.50 ± 3.85; *p* < 0.000001).

An analysis of BAEP showed the following significant abnormalities in patients in comparison with controls: prolonged latency of component V (5.85 ± 0.30 vs. 5.67 ± 0.19; *p* = 0.002), prolonged interpeak latencies III–V (1.95 ± 0.10 vs. 1.86 ± 0.16; *p* = 0.003) and I–V (4.12 ± 0.39 vs. 3.98 ± 0.19; *p* = 0.002), and lowered amplitude of components I (0.21 ± 0.08 vs. 0.30 ± 0.09; *p* = 0.00002) and V (0.33 ± 0.13 vs. 0.46 ± 0.16; *p* = 0.00047).

No significant differences in SEP parameters were found between the patients and controls.

In the patients, mean P300 latency was significantly longer than in the controls (Fz: 331.4 ± 29.5 vs. 316.1 ± 21.1; Cz: 335.1 ± 24.7 vs. 317.5 ± 20.7; Pz: 338.6 ± 22.4 vs. 320.3 ± 20.8; *p* = 0.001) and P300 amplitudes in Cz and Pz were significantly lower (Cz: 11.1 ± 4.6 vs. 9.0 ± 4.3; Pz: 11.0 ± 4.3 vs. 9.5 ± 4.1; *p* = 0.03).

In regard to age, a significant positive correlation was found for amplitude of N20/P22 SEP components (*R* = 0.42; *p* < 0.05) and amplitude of P300 in Fz (*R* = 0.36; *p* < 0.05). In comparison between the gender subgroups, women presented with higher mean amplitude of P100 (VEP) (11.8 vs. 7.8; *p* = 0.007), longer latency of N20 (18.9 vs. 20.2; *p* = 0.01), and shorter latency of the P16 component of SEP (16.04 vs. 17.2; *p* = 0.006).

#### Longitudinal Assessment

Table 3 presents analysis of the EP parameters evaluated at T0, T1, and T3.

**Table 3:** Comparison of EP parameters evaluated at T0, T1, and T3.

			**Mean** **±SD**			**Friedman test**	**Alpha with Bonferroni correction**
			**T0**	**T1**	**T3**	***p***	
**VEP**	**Latency (ms)**	N75	80.67 ± 11.89	78.81 ± 10.70	78.47 ± 11.06	0.394	0.0125
		P100	118.03 ± 11.75	111.49 ± 16.91	114.63 ± 11.3	0.040	
		N145	164.11 ± 16.51	159.84 ± 15.43	160.91 ± 17.7	0.901	
	**Amplitude (μV)**	P100/N145	10.90 ± 4.22	10.80 ± 5.12	10.19 ± 5.29	0.094	
**BAEP**	Latency (ms)	I	1.67 ± 0.10	1.66 ± 0.21	1.64 ± 0.19	*0.321*	0.0063
		III	3.88 ± 0.21	3.79 ± 0.45	3.77 ± 0.45	*0.632*	
		V	5.85 ± 0.27	5.74 ± 0.71	5.74 ± 0.68	*0.293*	
	Amplitude (μV)	I	0.20 ± 0.07	0.18 ± 0.07	0.18 ± 0.08	*0.538*	
		V	0.32 ± 0.11	0.35 ± 0.11	0.31 ± 0.13	*0.081*	
	**Interlatency (ms**)	I–III	2.22 ± 0.21	2.13 ± 0.27	2.13 ± 0.30	*0.610*	
		III–V	1.96 ± 0.11	1.95 ± 0.28	1.97 ± 0.27	*0.165*	
		I–V	4.15 ± 0.30	4.08 ± 0.53	4.09 ± 0.53	*0.834*	
**SEP**	**Latency (ms)**	N9	9.87 ± 0.71	9.89 ± 0.63	9.97 ± 0.60	*0.377*	0.005
		P10	11.54 ± 0.91	11.47 ± 0.66	11.52 ± 0.69	*0.767*	
		N13	13.30 ± 0.90	13.36 ± 0.72	13.81 ± 1.11	*0.016*	
		P16	16.50 ± 1.04	16.45 ± 0.92	16.54 ± 1.29	*0.186*	
		N20	19.49 ± 1.20	19.52 ± 0.94	19.67 ± 1.14	*0.245*	
		P22	22.45 ± 1.58	22.15 ± 1.11	22.30 ± 1.05	*0.152*	
	Interlatency	TT (N20–N13)	6.19 ± 0.48	6.16 ± 0.47	5.86 ± 0.91	*0.538*	
	**Amplitude (μV)**	N9/P10	3.02 ± 1.25	2.80 ± 1.24	2.56 ± 1.70	*0.212*	
		N13/P16	1.02 ± 0.37	0.90 ± 0.26	0.74 ± 0.32	0.003^*^	
		N20/P22	0.72 ± 0.41	0.61 ± 0.27	0.69 ± 0.45	*0.549*	
**ERP-300**	**Latency (ms)**	Fz	337.90 ± 27.77	335.33 ± 21.45	338.38 ± 25.70	*0.467*	0.0083
		Cz	338.55 ± 27.54	334.14 ± 23.58	337.52 ± 23.94	*0.405*	
		Pz	339.55 ± 26.33	320.62 ± 67.13	335.76 ± 30.24	*0.141*	
	**Amplitude (μV)**	Fz	9.43 ± 5.20	7.99 ± 5.43	8.31 ± 5.72	*0.229*	
		Cz	11.30 ± 4.57	10.19 ± 5.51	8.86 ± 4.44	*0.047*	
		Pz	10.33 ± 4.98	10.51 ± 4.90	9.23 ± 4.05	*0.368*	

* *significant after the Bonferrroni correction*.

***VEP** visual evoked potentials; **BAEP** brain auditory evoked potentials; **SEP** somatosensory evoked potentials; **ERP-300** the P300 event-related potentials*.

Mean N13/P16 amplitude in SEP was significantly lower at T1 and T3 than at baseline (*p* = 0.003). No other significant differences were found in the longitudinal comparison of EP results.

In addition, such longitudinal analysis of EP parameters was performed separately for the subgroups of patients who did or did not develop clinical or radiological indices of disease activity during the follow-up. There was no significant variation in EP parameters throughout T0, T1, and T3 for either of these subgroups.

#### Neuropsychological Testing—Longitudinal Assessment

**Figure 1:**
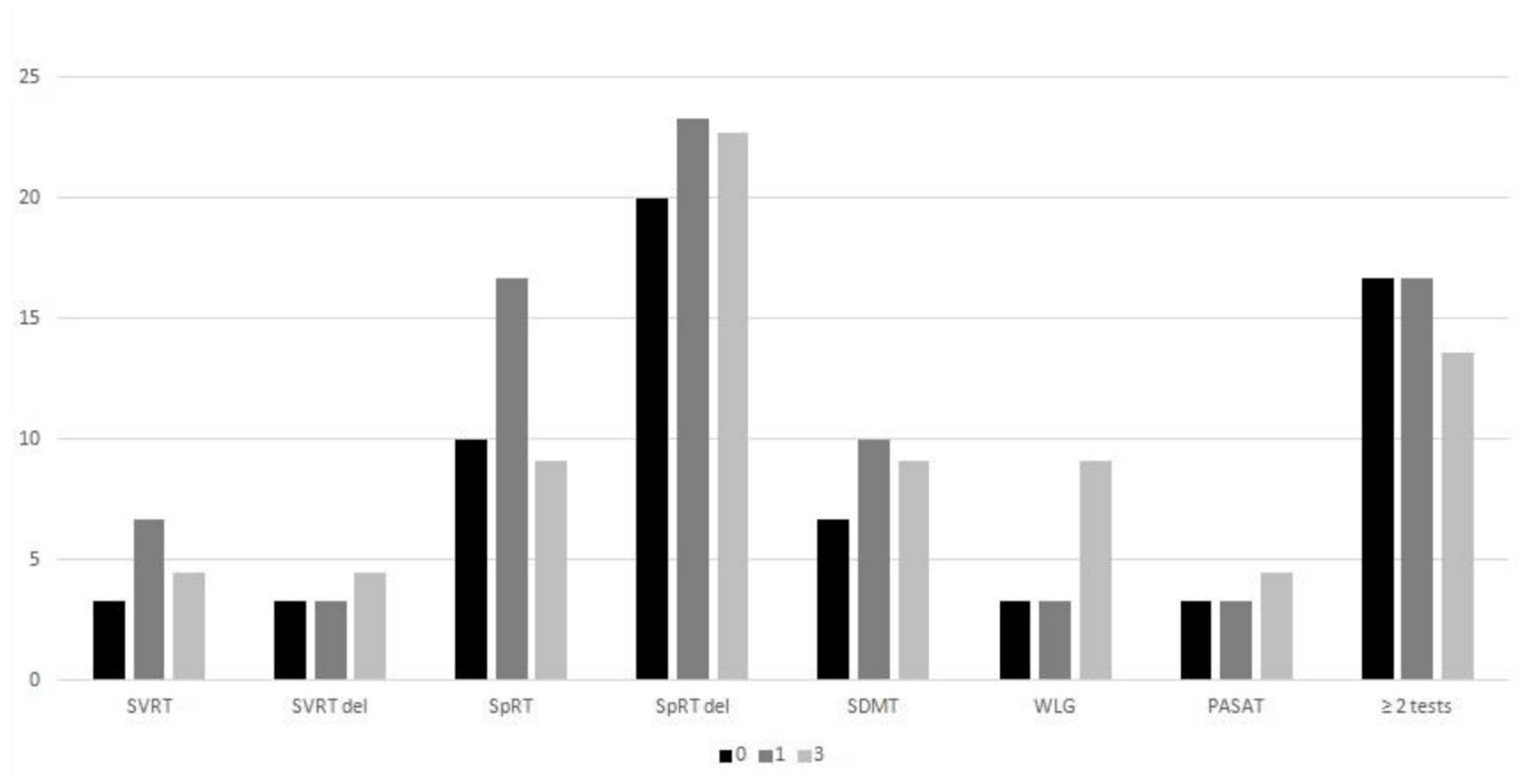
The results of BRBNT during the 3-year follow-up. BRBNT, Brief Repeatable Battery of Neuropsychological Tests.

Figure 1 shows the percentage of patients who failed in each of the tests from BRBNT and those who failed in at least two of the tests, at T0, T1, and T3. A trend was observed toward a smaller proportion of patients who failed in ≥2 tests in consecutive assessments, but without statistical significance (respectively, at T0, 17% of patients, at T1, 15% of patients, and at T3, 12% of patients).

Table 4 presents the mean results of tests included in BRBNT, assessed at T0, T1, and T3.

**Table 4:** Results of BRBNT in the study group at baseline and during the follow-up.

	**T0**	**T1**	**T3**
	**Mean ±SD**	**Mean ±SD**	**Mean ±SD**
FSS	2.81 ± 1.50	2.85 ± 1.35	3.15 ± 1.47
SVRT t	51.70 ± 6.96	51.47 ± 9.00	52.10 ± 9.39
SVRT del	8.73 ± 2.08	9.27 ± 1.89	9.14 ± 1.77
SpaRT	19.47 ± 5.78	22.13 ± 6.05	21.76 ± 4.81
SpaRT del	7.50 ± 2.37	7.93 ± 2.50	7.81 ± 2.36
SDMT	55.60 ± 10.30	56.47 ± 14.83	58.43 ± 13.38
WLG	26.60 ± 5.06	26.60 ± 4.30	26.19 ± 5.68
PASAT	52.00 ± 7.75	52.57 ± 7.07	52.24 ± 9.41

*BRBNT, Brief Repeatable Battery of Neuropsychological Tests; FSS, Fatigue Severity Scale; SVRT t, Selective Verbal Reminding Test total count of remembered words; SVRT del, Selective Verbal Reminding Test count of words recalled in a delayed part; SpaRT, Spatial Recall Test, SpaRT del, Spatial Recall Test delayed part of the test; SDMT, Symbol Digit Modalities Test; WLG, Word List Generation, PASAT, Paced Auditory Serial Additive Test*.

The mean result for SpaRT was significantly higher at T1 than at T0 (*p* < 0.05), but there was no significant difference for T3. No other significant changes in the results of BRBNT were found at each stage of follow-up.

In the baseline analysis of relationships between ERP parameters and BRBNT results, significant correlations were only found for P300 amplitude in Pz and SVRT t (*R* = 0.3; *p* = 0.036) and SDMT (*R* = 0.3; *p* = 0.041) scores. No correlations were analyzed between the respective findings from the evaluation at T1 and T3.

### Predictive Value of Baseline Findings

Table 5 shows the comparison of baseline EP and BRBNT results between the subgroups of patients who fulfilled the criteria for MS or remained in the CIS category, at each stage of the follow-up.

**Table 5:** Analysis of EP and BRBNT baseline parameters in the study group with regard to diagnosis of MS evaluated at T1 and T3.

			**T1**			**T3**		***p*-value**	**Alpha with Bonferroni correction**
			**CIS (16)**	**MS (13)**	**p-value**	**CIS (11)**	**MS (18)**		
**VEP**	**Latency (ms)**	N75	79.11	80.65	0.717	76.20	82.00	0.178	0.0125
		P100	115.70	115.63	0.988	112.43	117.65	0.247	
		N145	160.56	161.54	0.880	158.18	162.72	0.491	
	**Amplitude (μV)**	P100/N145	11.24	9.78	0.321	11.88	9.80	0.163	
**BAEP**	**Latency (ms)**	I	1.65	1.70	0.313	1.64	1.68	0.270	0.0063
		III	3.84	3.97	0.075	3.75	3.99	0.012^*^	
		V	5.78	5.94	0.145	5.67	5.96	0.007^*^	
	**Amplitude (μV)**	I	0.22	0.21	0.878	0.24	0.20	0.132	
		V	0.36	0.30	0.196	0.42	0.28	0.011^*^	
	Interlatency (ms)	I–III	2.15	2.27	0.203	2.05	2.30	0.015^*^	
		III–V	1.94	1.97	0.375	1.91	1.97	0.133	
		I–V	4.07	4.19	0.324	3.94	4.23	0.017^*^	
**SEP**	Latency (ms)	N9	9.75	9.93	0.535	9.67	9.93	0.397	0.005
		P10	11.29	11.65	0.312	11.19	11.61	0.243	
		N13	13.06	13.38	0.402	12.93	13.37	0.250	
		P16	16.42	16.45	0.625	16.43	16.44	0.763	
		N20	19.27	19.52	0.631	19.02	19.60	0.259	
		P22	22.07	22.58	0.401	21.58	22.72	0.065	
	Interlatency (ms)	TT	6.21	6.14	0.737	6.09	6.23	0.528	
	Amplitude **(μV)**	N9/P10	3.35	3.01	0.714	3.35	3.11	0.658	
		N13/P16	1.12	0.91	0.136	1.15	0.95	0.180	
		N20/P22	0.79	0.63	0.751	0.92	0.60	0.103	
**ERP** -P300	Latency (ms)	Fz	325.41	344.54	0.055	327.95	337.67	0.354	0.0083
		**Cz**	325.75	345.50	0.044^*^	327.64	338.86	0.277	
		**Pz**	325.56	352.69	0.001^**^	327.18	344.17	0.063	
	Amplitude **(μV)**	Fz	9.40	8.98	0.825	9.29	9.17	0.951	
		Cz	11.09	11.41	0.860	11.27	11.21	0.974	
		Pz	10.95	10.77	0.922	11.04	10.76	0.878	
**RBNT**		Vtotal	51.38	52.77	0.758	50.27	53.06	0.299	0.0071
		Vdel	9.00	8.62	0.625	8.27	9.17	0.263	
		SpRec3	19.19	19.92	0.982	20.00	19.22	0.736	
		SpRecdel	7.31	7.77	0.621	7.55	7.50	0.855	
		SymDig	57.69	52.92	0.230	57.91	54.11	0.242	
		Vflu	26.06	27.62	0.401	25.18	27.72	0.196	
		PASAT	53.69	51.23	0.369	52.82	52.44	0.718	

* *Not significant after Bonferrroni correction*.

** *Statistically significant after Bonferroni correction*.

***EP** visual evoked potentials; **BAEP** brain auditory evoked potentials; SEP, somatosensory evoked potentials; **ERP-300** the P300 event-related potentials; **RBNT** Brief Repeatable Battery of Neuropsychological Tests*.

The patients classified as MS at T1 had at longer baseline mean P300 latency in Cz and Pz than those still assigned as CIS. Those classified as MS at T3, in comparison with the subgroup with CIS, had at longer baseline latency of III and V components, prolonged interlatencies I–III and I–V, and reduced amplitude wave V of BAEP.

After Bonferroni correction for multiple comparisons had been applied. The only significant difference (between those classified as MS or CIS at T1) was maintained for the P300 latency in Pz. Baseline results of BRBNT did not differentiate patients with MS and those with CIS at any of the analyzed time points.

Baseline EP parameters were also compared between the subgroups of patients differing in changes in EDSS rating from T0 to T3 (Table 6). After using Bonferroni correction for multiple comparisons, no significant differences in baseline EP measures were found between the patients with increased or stable EDSS score during the follow-up from T0 to T3.

**Table 6:** Comparison of baseline EP parameters between the subgroups of patients with increased or stable EDSS during 3 years of follow-up.

			**Mean** **±SD**		***p*-value**	**Alpha with Bonferroni correction**
			**EDSS T3/T0 stable (*n* = 21)**	**EDSS T3/T0 increased (*n* = 8)**		
**VEP**	**Latency (ms)**	N75	79.56 ± 10.55	80.44 ± 13.31	0.853	0.0125
		P100	115.69 ± 10.94	115.63 ± 14.06	0.989	
		N145	162.62 ± 18.02	156.75 ± 13.30	0.411	
	**Amplitude (μV)**	P100/N145	10.07 ± 4.00	11.96 ± 3.34	0.244	
**BAEP**	Latency (ms)	I	1.68 ± 0.11	1.63 ± 0.10	0.107	0.0063
		III	3.96 ± 0.24	3.73 ± 0.22	0.017	
		V	5.92 ± 0.28	5.67 ± 0.29	0.044	
	Amplitude **(μV)**	I	0.21 ± 0.08	0.23 ± 0.08	0.453	
		V	0.31 ± 0.13	0.40 ± 0.12	0.064	
	**Interlatency (ms)**	I–III	2.25 ± 0.32	2.10 ± 0.18	0.067	
		III–V	1.95 ± 0.09	1.94 ± 0.13	0.801	
		I–V	4.15 ± 0.44	4.05 ± 0.25	0.188	
**SEP**	**Latency (ms)**	N9	9.97 ± 0.71	9.50 ± 0.75	0.136	0.005
		P10	11.65 ± 0.89	10.98 ± 0.79	0.077	
		N13	13.47 ± 0.94	12.58 ± 0.69	0.024	
		P16	16.67 ± 1.03	15.88 ± 1.07	0.094	
		N20	19.70 ± 1.26	18.62 ± 1.01	0.041	
		P22	22.63 ± 1.63	21.51 ± 1.04	0.086	
	Interlatency	TT (N20–N13)	6.24 ± 0.55	6.04 ± 0.53	0.392	
	**Amplitude (μV)**	N9/P10	3.12 ± 1.45	3.40 ± 0.78	0.609	
		N13/P16	1.04 ± 0.36	1.00 ± 0.38	0.795	
		N20/P22	0.65 ± 0.48	0.88 ± 0.54	0.353	
**ERP**-P300	**Latency (ms)**	Fz	335.40 ± 24.78	330.25 ± 33.25	0.652	0.0083
		Cz	337.02 ± 24.06	328.25 ± 33.12	0.436	
		Pz	339.98 ± 18.89	331.81 ± 35.03	0.423	
	**Amplitude (μV)**	Fz	9.46 ± 4.41	8.57 ± 6.62	0.679	
		Cz	11.38 ± 4.64	10.86 ± 5.35	0.800	
		Pz	10.87 ± 4.71	10.85 ± 5.20	0.988	

***VEP** visual evoked potentials; **BAEP** brain auditory evoked potentials; **SEP** somatosensory evoked potentials; **ERP-300** the P300 event-related potentials*.

In addition, a logistic regression model was used to assess the predictive value of baseline EP and BRBNT results in regard to conversion from CIS to MS. None of the analyzed variables were demonstrated to have a significant predictive value for the outcome at T1 or T3. Logistic regression against NEDA-3 status showed that the baseline values of evoked potential parameters (VEP, BAEP, SEP, ERP) and results of psychological tests were not predictive for clinical or radiological indices of disease activity during the follow-up. Logistic regression failed to identify any predictor. Also, when the location of demyelinating foci on MR examination was analyzed, taking into account the division into a group with supratentorial lesions only and a subgroup with supra- and infra-tentorial lesions, no predictive factor could be identified in logistic regression.

## Discussion

### Baseline Assessment

An occult damage to CNS occurs prior to the first clinical manifestation of MS, recognized as CIS. MRI or EP findings may reveal silent lesions, which do not correspond with clinical symptoms and provide evidence for dissemination in space. In the study group, most common manifestations of CIS included optic neuritis and sensory disturbances, with fewer frequent brainstem and pyramidal symptoms. At the baseline assessment of EP, the latency of VEP was indeed increased, while parameters of BAEP were significantly more affected than SEP. Because of the relatively small sample size, we did not divide the subjects into subgroups according to their clinical presentation, for separate analysis of EP parameters.

BAEP are considered to have low sensitivity due to the shortest pathway explored (5), but their capacity to reveal subclinical brainstem involvement seems relevant. Some authors (6, 17, 20) suggested the use of additional modalities (vestibular myogenic EP, tongue SEP) to improve the evaluation of brainstem function at the early stage of MS. A selection of modalities for an optimal EP protocol remains a matter of debate. SEP and MEP, especially from lower limbs, are regarded as sensitive measures of long corticospinal pathways integrity (5, 12). Our EP protocol was designed to cover the functionality of sensory pathways, and SEP were recorded from upper limbs only, to reduce the burden of testing and emerging fatigue in the patients. We also decided to extend the multimodal approach, including ERP—a measure of cognitive performance, an important aspect of “invisible disability.” The findings from our previous report (13) and a few other studies (21, 22) confirmed the usefulness of ERP, accompanied by neuropsychological testing, in evaluation of early cognitive impairment in patients with CIS. Although few correlations were found between ERP parameters and BRBNT results, electrophysiological and neuropsychological markers of cognitive performance are considered to be complementary to each other and presumably cover different aspects of cognitive processes (21, 22). To the best of our knowledge this is the first attempt to include event-related potentials into serial multimodal EP analysis, within prospective observation of the patients at the earliest stage of MS.

### Longitudinal Assessment

EP have been investigated for their potential use in monitoring the course of MS and a response to treatment, and it was suggested that the longitudinal change in EP scores over time may reflect the activity of disease (12). In our study, except for lowered amplitude of the SEP cortical component, no significant longitudinal changes in EP parameters were found over the 3 years either for the whole group, or for the separated subgroup with clinical or radiological signs of disease activity. Possible contributing factors may include fluctuating and potentially reversible disturbances in the functionality of neural pathways as well as individual variability of EP parameters over time. Other studies based on serial multimodal EP assessment in MS subjects (7–9) focused on the relationships between cumulative EP score and EDSS and found a moderate correlation between their longitudinal changes within 1–5 years of follow-up. The prospective evaluation of ERP (23, 24) showed lowered amplitudes and prolonged latencies of P300 within short (1 year) or long-term (8 years) observation.

Similarly to ERP parameters, BRBNT results in the studied group did not change significantly in longitudinal assessments over the 3 years. There was a trend toward a smaller proportion of patients who failed ≥2 tests, but without statistical significance. At the early stage of MS, some authors have reported a stable level of cognitive performance (25–27), while others have observed an improvement (23, 28, 29) or deterioration (21, 30). Practice effect has to be taken into account in interpretation of such serial findings, but BRBNT is regarded as test-retest reliable and represents a compromise practice effect across the tests (31).

It should be also highlighted that the majority of our patients were being treated with DMT during the follow-up. Single or multimodal EP parameters were reported as a possible marker for response to treatment with IFN β (32) and fingolimod (33). The studies analyzing the impact of IFN β upon ERP parameters showed conflicting results: either no significant ERP differences (28), or a reduction in P300 latencies and a trend toward a reduction of amplitudes (29) during treatment. The heterogeneity of our group (including diverse duration of treatment, discontinuation or switch of DMT) did not enable us to precisely evaluate the effect of treatment response upon electrophysiological and neuropsychological measures.

### Predictive Value of Baseline Findings

Out of 29 subjects initially diagnosed with CIS, 10 patients retrospectively fulfilled the current McDonalds criteria for MS, 8 converted from CIS to MS within 3 years of follow-up, and 11 still remained in the CIS category after 3 years. On comparative analysis of their baseline findings, we found that prolonged latency of P300 was associated with conversion to MS within the first year (and this finding maintained significance after additional analysis for multiple comparisons). Thus electrophysiological measures of cognitive performance (unlike neuropsychological testing results) seemed to be associated with short-term activity of the disease. In other prospective studies including patients with CIS (10, 11), multimodal EP abnormalities were found to predict earlier conversion from CIS to MS (with clinical and/or radiological signs of dissemination in time), independently from MRI or CSF baseline findings. Pelayo et al. (34) demonstrated that individual EP scores did not significantly affect the risk of conversion, and the predictive value of multiple EP abnormalities was limited by their small proportion. In the retrospective stratification of risk factors for conversion from CIS into MS (35), neither single nor overall EP score showed significant predictive value.

Prognostic power of EP was more frequently analyzed with regard to progression of disability. In retrospective studies, baseline scores of multimodal EP correlated with sustained accumulation of disability (6) or achievement of EDSS milestones within a few years (8). Several prospective reports showed that abnormalities of multimodal EP predicted progression of disability during short (2 years) or long-term (5–10 years) follow-up (7, 9, 11, 34, 36). However, these relationships were considered as more relevant for relapsing-remitting or progressive MS than for CIS as the earliest stage of disease (12). Although various combinations of modalities and cumulative scores were used in the cited studies. SEP and MEP from lower limbs as well as brainstem-related EP most consistently showed correlation with EDSS during follow-up (6, 8). On the contrary to these reports, we found no differences in baseline EP parameters between the patients with stable EDSS over three years of follow-up and those whose disability level increased. It should be considered that overall disability level in the study group was low (mean EDSS 1.5 after 3 years), presumably as a result of mild relapses and complete remissions. Moreover, a small percentage of patients with an increase in EDSS score could have affected the reliability of analysis.

In regard to cognitive measures in CIS, some authors have found baseline results of neuropsychological testing to be predictive for conversion into MS as well as for further cognitive decline (37, 38). However, there is no available evidence for the prognostic value of ERP parameters, so our findings on P300 abnormalities suggest they deserve attention in this field.

Analysis of baseline electrophysiological and neuropsychological findings with regard to disease outcomes in our study, using a logistic regression model, failed to demonstrate a significant predictive value for any of the investigated variables. The power of these findings was presumably affected by the relatively small sample size, which has to be addressed as a serious limitation to this study. Originally, the study group comprised 44 subjects diagnosed with CIS, which was considered representative for the tertiary reference neurological center in our country. However, as some of these patients resigned from further follow-up (which is a common problem of prospective studies), only 29 patients were available for complete longitudinal analysis. Another limitation was associated with clinical heterogeneity of the study group which would require separate analysis of the relevant subgroups, not eligible due to the small number of patients.

However, our findings hopefully provide a better insight into various aspects of neurological deficit (including impaired cognition) at the earliest stage of MS and contribute to a discussion on the role of EP as electrophysiological markers of MS-related CNS dysfunction. Further investigation might include the optimal choice of modalities for EP protocol and identification of patients who would benefit most from the use of EP as diagnostic and monitoring tools.

## Conclusions

Baseline ERP abnormalities were associated with their conversion into MS in short-term observation. In longitudinal assessment, EP and neuropsychological testing did not provide a measure for activity or progression of the disease. ERP, as electrophysiological markers of cognitive performance, are worth considering in multimodal EP evaluation in patients at the early stage of MS.

## Data Availability Statement

The original contributions presented in the study are included in the article/supplementary material, further inquiries can be directed to the corresponding author.

## Ethics Statement

The studies involving human participants were reviewed and approved by the Bioethics Committee of Medical University and complied with the ethical standards required. The patients/participants provided their written informed consent to participate in this study.

## Author Contributions

ED and AP-D: study concept, design, and critical revision of the manuscript for important intellectual content. MW: statistical analysis. ED, MZ, EG, and JC-Ł: data collection. ED and EG: drafting the manuscript. ED, SB, and AP-D: data analysis and interpretation. All authors contributed to the article and approved the submitted version.

## Funding

This work was supported by Wroclaw Medical University SUB.C.220.19.056.

## Conflict of Interest

The authors declare that the research was conducted in the absence of any commercial or financial relationships that could be construed as a potential conflict of interest.

## Publisher's Note

All claims expressed in this article are solely those of the authors and do not necessarily represent those of their affiliated organizations, or those of the publisher, the editors and the reviewers. Any product that may be evaluated in this article, or claim that may be made by its manufacturer, is not guaranteed or endorsed by the publisher.

## Abbreviations

BAEP: Brainstem auditory evoked potentials
BRBNT: Brief Repeatable Battery of Neuropsychological Tests
CNS: central nervous system
CIS: clinically isolated syndrome
DMT: disease-modifying treatment
ERP: event-related potentials
EP: evoked potentials
EDSS: Expanded Disability Status Scale
NT: neuropsychological tests
NEDA: No Evidence of Disease Activity
MS: multiple sclerosis
PASAT: Paced Auditory Serial Additive Test
SVRT: Selective Verbal Reminding Test
SEP: somatosensory evoked potentials
SpaRT: Spatial Recall Test
SDMT: Symbol Digit Modalities Test
VEP: visual evoked potentials
WLG: Word List Generation.
